# Multi-Agent Chemotherapy Overcomes Glucocorticoid Resistance Conferred by a *BIM* Deletion Polymorphism in Pediatric Acute Lymphoblastic Leukemia

**DOI:** 10.1371/journal.pone.0103435

**Published:** 2014-08-04

**Authors:** Sheila Xinxuan Soh, Joshua Yew Suang Lim, John W. J. Huang, Nan Jiang, Allen Eng Juh Yeoh, S. Tiong Ong

**Affiliations:** 1 Cancer and Stem Cell Biology Program, Duke-NUS Graduate Medical School, Singapore, Singapore; 2 Department of Paediatrics, Yong Loo Lin School of Medicine, National University of Singapore, Singapore, Singapore; 3 National University Cancer Institute, National University Health System, Singapore, Singapore; 4 Viva-University Children's Cancer Centre, University Children's Medical Institute, National University Health System, Singapore, Singapore; 5 Cancer Science Institute, National University of Singapore, Singapore, Singapore; 6 Department of Haematology, Singapore General Hospital, Singapore, Singapore; 7 Department of Medical Oncology, National Cancer Centre, Singapore, Singapore; 8 Division of Medical Oncology, Duke University Medical Center, Durham, North Carolina, United States of America; University of Sydney, Australia

## Abstract

A broad range of anti-cancer agents, including glucocorticoids (GCs) and tyrosine kinase inhibitors (TKIs), kill cells by upregulating the pro-apoptotic BCL2 family member, BIM. A common germline deletion in the *BIM* gene was recently shown to favor the production of non-apoptotic BIM isoforms, and to predict inferior responses in TKI-treated chronic myeloid leukemia (CML) and EGFR-driven lung cancer patients. Given that both *in vitro* and *in vivo* GC resistance are predictive of adverse outcomes in acute lymphoblastic leukemia (ALL), we hypothesized that this polymorphism would mediate GC resistance, and serve as a biomarker of poor response in ALL. Accordingly, we used zinc finger nucleases to generate ALL cell lines with the *BIM* deletion, and confirmed the ability of the deletion to mediate GC resistance *in vitro*. In contrast to CML and lung cancer, the *BIM* deletion did not predict for poorer clinical outcome in a retrospective analysis of 411 pediatric ALL patients who were uniformly treated with GCs and chemotherapy. Underlying the lack of prognostic significance, we found that the chemotherapy agents used in our cohort (vincristine, L-asparaginase, and methotrexate) were each able to induce ALL cell death in a BIM-independent fashion, and resensitize *BIM* deletion-containing cells to GCs. Together, our work demonstrates how effective therapy can overcome intrinsic resistance in ALL patients, and suggests the potential of using combinations of drugs that work via divergent mechanisms of cell killing to surmount *BIM* deletion-mediated drug resistance in other cancers.

## Introduction

Genome-wide profiling studies of acute lymphoblastic leukemia (ALL) have revealed it to be a highly heterogeneous disease [1]. In spite of this, the majority of ALL subtypes are treated with a remission-induction protocol that invariably consists of a glucocorticoid, vincristine and at least one other chemotherapy agent (L-asparaginase, an anthracycline, or both) [2]. Unfortunately, 15-20% of patients continue to relapse, and outcome remains poor for these individuals [3]. Consequently, there have been ongoing efforts to identify genetic factors that could account for this response heterogeneity and serve as prognostic markers for risk stratification or novel druggable targets in order to improve patient outcomes [4]–[6].

At the same time, recent reviews have underscored the notion that response heterogeneity can arise from not only somatic mutations but also germline polymorphisms [7], [8]. A number of examples of the latter have been described, including genetic variants that influence the pharmacokinetic and pharmacodynamic phenotype of the host, as well as those affecting the underlying biology of the leukemic cell and thereby cell intrinsic drug resistance/sensitivity [9]–[15]. Notably, however, studies correlating genetic variants with clinical phenotypes have been largely based on genetic epidemiology data and lack experimental validation at a mechanistic level. Such mechanistic studies have been hampered in part by the difficulty and cost of generating isogenic cell lines that either possess or lack a mutation of interest. More recently, a variety of methods that enable genome engineering to faithfully recapitulate mutations of interest have been developed and these will aid the functional validation of these variants *in vitro* [16].

Using such an approach, we recently validated the functional consequences of a germline deletion in the *BIM* gene in chronic myeloid leukemia (CML) [17]. Unlike in ALL, a single causative lesion, the 9;22 translocation, is known to be present in >95% of chronic myeloid leukemia (CML) cases [18]. Despite the targeted nature of tyrosine kinase inhibitors (TKIs), response heterogeneity is also a significant challenge in CML [19]. From a group of TKI-resistant CML patients, we identified a 2.9 kb intronic deletion in the *BIM* gene, and later verified it to be a polymorphism found in 12.3% of East Asians [17]. *BIM* encodes a potent pro-apoptotic BH3-only protein that is required for specific anti-cancer therapies to induce apoptotic cell death [20]–[25]. When we introduced the deletion into a CML cell line using zinc finger nuclease-based technology, the polymorphism was sufficient to cause intrinsic resistance to tyrosine kinase inhibitors. Mechanistically, we showed that the *BIM* deletion biases splicing toward BIM isoforms that lack the BH3 domain encoded in exon 4, resulting in the expression of BIM isoforms incapable of inducing apoptosis. Consistent with the *in vitro* data, both CML and EGFR-driven lung cancer patients carrying the polymorphism experienced inferior responses to treatment with tyrosine kinase inhibitors.

Since BIM is required for GC-induced apoptosis in lymphoid lineage cells, including ALL cells [26]–[32], and both *in vitro* and *in vivo* GC response has been shown to predict favorable treatment outcome in ALL [33]–[37], we wondered if the polymorphism could contribute to response heterogeneity in ALL patients. If this were the case, we expect that pharmacological restoration of BIM function using drugs such as BH3 mimetics would enable us to improve response in patients with the polymorphism [17], [25]. Furthermore, because multi-agent chemotherapy is essential to the long-term control of pediatric ALL, the clinical model of ALL could allow us to determine the interaction between a single germline variant and combination therapy.

Accordingly, we used zinc finger nucleases to generate *de novo* ALL cell lines with the *BIM* deletion polymorphism in both heterozygous and homozygous configurations. Using these lines, we found that the *BIM* deletion polymorphism was sufficient to confer GC resistance *in vitro*. However, analysis of a pediatric ALL cohort uniformly treated with GCs and chemotherapy [38] revealed that patients with the *BIM* deletion did not experience inferior response rates nor poorer clinical outcomes. Mechanistically, we determined that GC resistance conferred by the *BIM* polymorphism could be overcome with the addition of chemotherapeutic agents used in standard ALL protocols, and which likely act via a BIM-independent mechanism to cause cell death. Together, our data demonstrate that, whilst the *BIM* deletion is sufficient to confer resistance to GCs, the negative impact of polymorphic variants on single agent therapy can be overcome with multi-agent chemotherapy that kill cancer cells via divergent mechanisms. These results highlight the challenge of identifying genetic markers predictive of clinical outcome in populations treated with multi-agent therapy, the utility of genome editing technologies in the study of polymorphic variants, as well as the importance of using drug combinations that kill cancer cells via non-overlapping mechanisms.

## Methods

### Cell lines and culture conditions

CCRF-CEM was purchased from the American Type Culture Collection (Manassas, VA, USA). Cells were maintained in RPMI-1640 media (Nacalai Tesque, Japan) supplemented with 20% FBS, penicillin/streptomycin and L-glutamine (all from Thermo Scientific, Rockford, IL, USA). Dexamethasone (Rotexmedica, Germany), methotrexate (ABIC Ltd, Israel), vincristine (Korea United Pharm Inc, Korea) and L-asparaginase (Kyowa Hakko Kirin, Japan) were used at the dosages and times indicated in the figure legends. All experiments using cell lines were performed at least 3 times.

### Creation of genome-edited lines

The zinc finger nucleases (ZFNs) targeting the *BIM* gene were custom-made (Sigma-Aldrich, St Louis, MO, USA) and the repair template was generated as described in a previous paper [17]. Plasmids encoding the repair template and ZFNs were transfected into CCRF-CEM cells using the Neon system (Invitrogen, Carlsbad, CA, USA). Clones were isolated by dilution cloning and screened for presence of the deletion using the following primers: Forward (5′-GGCCTTCAACCACTATCTCAGTGCAATGG-3′) and Reverse (5′- GGTTTCAGAGACAGAGCTGGGACTCC-3′). qPCR to determine exon 3 to exon 4 ratio was performed as previously described [17].

### MTS assays

Cells were seeded at a density of 4×10^4^ per well in a 96-well plate and incubated with the indicated drugs. In each experiment, every treatment condition was repeated in triplicate wells. After 48 h, CellTiter AQueous One Solution Cell Proliferation reagent (Promega, Fitchburg, WI, USA) was added to each well and incubated for 2 h before an absorbance reading at 490 nm was taken.

### Immunoblotting

Cells were washed once in PBS and lysed in RIPA lysis buffer (Millipore, Billerica, MA, USA) containing proteinase inhibitor (Roche, Indianapolis, IN, USA). Protein concentrations were assayed using the Quick Start Bradford protein assay kit (Bio-Rad) and bovine serum albumin as a standard. The following antibodies were used at these concentrations: phospho-glucocorticoid receptor (S211) (Cell Signaling Technology, Danvers, MA, USA #4161, 1∶1 000), glucocorticoid receptor (BD Transduction Laboratories, San Jose, CA, USA, 1∶2 000), β-actin (Sigma-Aldrich, 1∶10 000), PARP (Cell Signaling Technology #9542, 1∶2 000), caspase 3 (Cell Signaling Technology #9663, 1∶500) and BIM (Cell Signaling Technology #2819, 1∶1 000). HRP-conjugated secondary antibodies against mouse or rabbit IgG (Santa Cruz Biotechnology, Santa Cruz, CA, USA) were used at 1∶10 000. Western Lightning ECL reagent (PerkinElmer, Waltham, MA, USA) was used to visualize the protein bands. Any adjustments to contrast and intensity were applied uniformly to the images.

### Patient recruitment

411 patients with newly-diagnosed ALL from the Malaysia-Singapore (Ma-Spore) acute lymphoblastic leukemia (ALL) 2003 study [38] were included on the basis of DNA availability. Written informed consent was obtained from the parents or the legal guardians of the patients. The study was approved by the National Healthcare Group Domain Specific Review Board (NHG DSRB). Since the *BIM* deletion is germline in nature, and will be present in both normal and leukemic samples, we were able to employ both remission (n = 362) and diagnostic (n = 49) samples for genotyping for this study. Patient risk stratification, details of the treatment protocol, minimal residual disease monitoring and molecular subgrouping were described previously [38].

### Statistical analyses

Statistical analysis was performed using SPSS software (version 16.0 for Windows; IBM Corporation, Armonk, NY, USA). Comparisons between groups were examined by Fisher's exact test for categorical variables. A statistically significant difference was defined as a *P* value of <0.05. Survival curves were evaluated using Kaplan–Meier analysis. Event-free survival (EFS) was defined as the time from diagnosis to first recurrence of the disease, including induction failure, or death. Induction failure was defined as failure to achieve complete remission and considered as an event at one day after date of diagnosis. Overall survival (OS) was defined as the time from diagnosis to death. Patients who were alive and had no progression of disease or relapse were censored at the time of their last follow-up.

## Results

### *De novo* generation of ALL cell lines bearing the *BIM* deletion polymorphism

**Figure 1:**
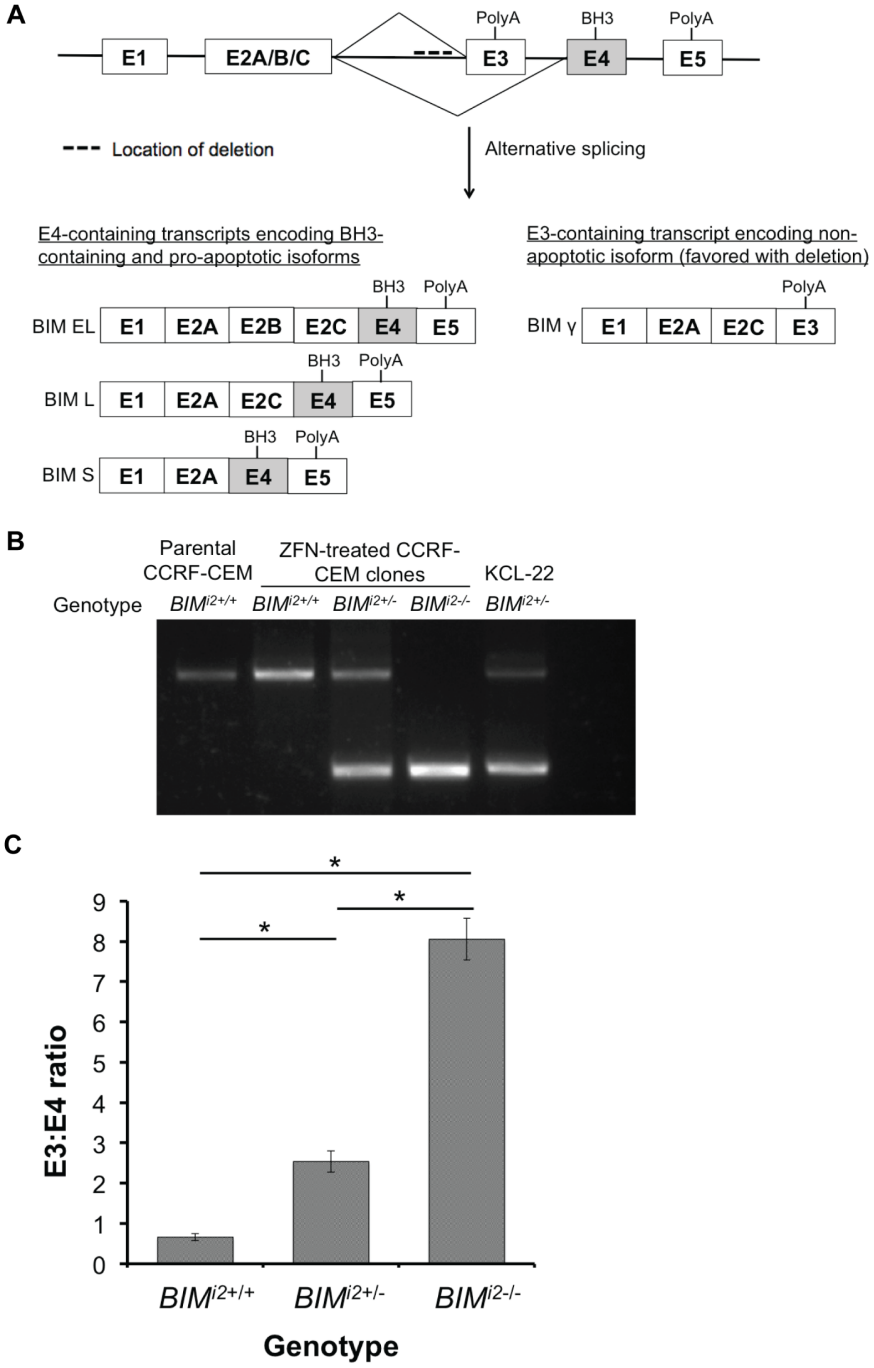
Generation of isogenic CCRF-CEM cell lines with the *BIM* deletion polymorphism. (**A**) Structure of the *BIM* gene and major splice isoforms. The *BIM* deletion polymorphism lies within intron 2 and upstream of exon 3, as indicated by the dashed line. Exon 4 contains the crucial BH3 domain required for apoptosis. Exon 3 (E3) and 4 (E4) are spliced in a mutually exclusive fashion, leading to the generation of either E4-containing isoforms with the BH3 domain (BIM EL, BIM L and BIM S) or E3-containing isoforms without the BH3 domain (BIM γ). When present, the deletion biases splicing towards E3-containing non-apoptotic isoforms. (**B**) Agarose gel of the products from a PCR reaction to detect the polymorphism in zinc finger nuclease (ZFN)-treated CCRF-CEM subclones, with the lower band indicating the presence of the deletion. Parental CCRF-CEM and KCL-22 cells (a CML cell line known to be heterozygous for the *BIM* deletion polymorphism) were included as controls. (**C**) The ratio of exon 3 to exon 4-containing transcripts (E3:E4) in CCRF-CEM *BIM^i2+/+^*, *BIM^i2+/−^* and *BIM^i2−/−^* clones as measured by qPCR. Error bars indicate mean ± SEM (n = 3). A student's t-test was performed for pairwise comparisons of E3:E4 ratio between genotypes. * indicates a significant difference with *P*<0.05.

To determine if the *BIM* deletion polymorphism is sufficient to confer GC resistance to ALL cells, we used zinc finger nucleases to derive *de novo* ALL cell lines bearing the deletion. Because human cell lines vary in their amenability to transfection and genome editing by zinc fingers (personal communication, TK Ko and unpublished observations), we tested the ability of our approach to edit 3 different GC-sensitive ALL cell lines (CCRF-CEM, RS4;11, and PALL-2) [39]–[41] that did not have the polymorphism. Of these lines, we were only able to successfully generate clones containing the deletion in CCRF-CEM cells. The structure of the *BIM* gene and the location of the deletion in intron 2 are illustrated in Figure 1A. Using PCR primers that flank the deleted region, we identified subclones that either did not have the deletion (denoted *BIM^i2+/+^*) or were heterozygous (denoted *BIM^i2+/−^*) or homozygous (denoted *BIM^i2−/−^*) for the deletion polymorphism (Figure 1B). The deleted region contains splicing elements that either promote the production of functional, exon 4-containing isoforms or suppress the production of non-apoptotic, exon 3-containing isoforms [42]. Consequently, when deleted, an increase in exon 3 to exon 4-containing transcripts is expected. To confirm that the deletion produced the expected changes in the splicing of *BIM*, we measured the ratio of exon 3- to exon 4-containing transcripts in clones of each genotype by exon-specific RT-PCR, and found that it was increased in a polymorphism-dosage-dependent manner (Figure 1C).

### The *BIM* deletion polymorphism is sufficient to confer GC resistance in ALL cells

**Figure 2:**
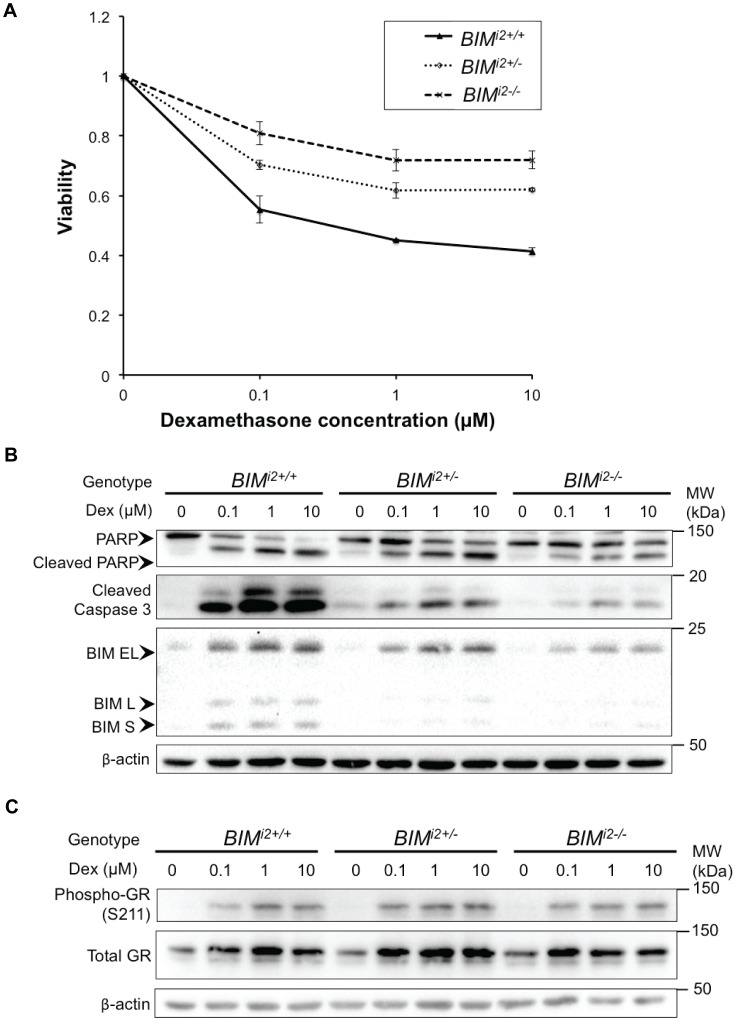
The *BIM* deletion confers dexamethasone resistance in CCRF-CEM cells. (**A**) Cell viability following exposure of CCRF-CEM subclones to increasing concentrations of dexamethasone. Viability was measured by MTS assay at 48 h. Error bars indicate mean ± SEM (n = 3) of 3 independent replicates. (**B**) Western blot of cell lysates from CCRF-CEM *BIM^i2+/+^*, *BIM^i2+/−^* and *BIM^i2−/−^* clones following treatment with increasing doses of dexamethasone for 48 h. The induction of cleaved PARP and cleaved caspase 3 were used as readouts for apoptosis. An antibody that recognizes pro-apoptotic exon-4 containing BIM isoforms (BIM EL, L and S) was used to show the extent of BIM upregulation following GC exposure. β-actin was used as a loading control. (**C**) Western blot showing phosphorylation of the glucocorticoid receptor (Phospho-GR S211) in CCRF-CEM *BIM^i2+/+^*, *BIM^i2+/−^* and *BIM^i2−/−^* clones upon treatment with dexamethasone.

To determine if the deletion conferred resistance to GCs, we compared the effect of treating clones of each genotype with a range of dexamethasone concentrations. First, we quantified cell viability using the MTS assay and found that across the range tested, the deletion-containing clones exhibited increased cell viability in a polymorphism dosage-dependent manner (Figure 2A). Following this, we assessed the extent of apoptosis using the induction of cleaved poly ADP-ribose polymerase (PARP), as well as the level of cleaved caspase 3, using immunoblots performed on lysates of cells treated as in Figure 2A. We also probed for BIM using an antibody that only detects the pro-apoptotic E4-containing isoforms (BIM EL, L, and S). As a marker of glucocorticoid receptor (GR) activation, we probed for phospho-GR (S211). As predicted, when compared to wildtype clones, upregulation of E4-containing BIM isoforms was impaired in a polymorphism dosage-dependent manner. Furthermore, apoptosis was attenuated in clones with the deletion, as evidenced by an increase in cleaved PARP, as well as cleaved caspase 3 (Figure 2B). Importantly, this occurred in spite of equivalent GR phosphorylation and auto-induction upon GC treatment across the genotypes (Figure 2C). These results indicate that GC resistance in the deletion-containing clones takes place downstream of the GR, and is consistent with our hypothesis that GC resistance results from impaired expression of BH3-containing BIM isoforms. Taken together, our data demonstrate that the presence of the *BIM* deletion polymorphism is sufficient to confer GC resistance in ALL cells.

Because prior work has shown that *in vitro* GC responses *per se* is an important prognostic factor in childhood ALL [33], [34], we predicted that patients with the *BIM* deletion polymorphism would have inferior outcomes compared to those without.

### The *BIM* deletion polymorphism does not predict inferior responses in pediatric ALL

To test our prediction that the *BIM* deletion polymorphism confers a poorer clinical outcome in pediatric ALL, we conducted a retrospective analysis correlating treatment outcome with the presence of the polymorphism in a group of uniformly-treated pediatric patients from the Malaysia-Singapore (Ma-Spore) ALL 2003 multicenter study. The Ma-Spore ALL 2003 protocol was based on a modified Berlin-Frankfurt-Münster regimen, where all patients received intrathecal methotrexate together with seven days of oral prednisolone at the point of diagnosis. Patients subsequently completed the rest of their induction regimen based on a common backbone of vincristine, L-asparaginase, and methotrexate, followed by risk-adapted consolidation and maintenance therapy as directed by their MRD status at day 33. Importantly, the design of this study allowed us to determine if the *BIM* deletion predicts for inferior clinical outcomes at three distinct assessment points: initial GC response (defined as absolute blast count ≥1000/µl at day 8), day 33 MRD following multi-agent induction chemotherapy, as well as overall survival (OS) and event-free survival (EFS) after consolidation and maintenance therapy.

Sufficient DNA from 411 individuals (out of a total of 556) from the Ma-Spore study was available for analysis for the *BIM* deletion polymorphism. Importantly, there was no difference in treatment outcome between this subgroup of 411 patients compared to the 556 patients in the full study (5-year EFS 82.0% vs 80.6%). Using this sample set, we determined the incidence of the *BIM* deletion to be 12.2%, which is consistent with the ethnic make up of the Ma-Spore cohort, as well as the incidence of the polymorphism in the normal population (Table 1, [17]). We also found that the *BIM* deletion polymorphism did not segregate according to any patient demographic except for Chinese ethnicity, which is as expected, or adverse prognostic indicators such as genetic subtype (Table 1).

**Table 1:** Biological and clinical features of patients from the Ma-Spore ALL 2003 trial genotyped for the *BIM* polymorphism.

Characteristics					*P* value
	Wildtype (n = 361)		*BIM* polymorphism present (n = 50)		
	No. %		No. %		
Age at diagnosis					1.000
<1 or >10	71	19.7	10	20	
1–10	290	80.3	40	80	
Sex					0.094
Male	213	59	23	46	
Female	148	41	27	54	
Molecular subtype∧					0.127
*ETV6-RUNX1*	68	19	7	14.3	
* TCF-PBX1*	17	4.7	7	14.3	
* BCR-ABL1*	16	4.5	1	2	
MLL rearrangements	9	2.5	3	6.1	
Hyperdiploidy	65	18.2	12	24.5	
Hypodiploidy	4	1.1	0	0	
T-ALL	30	8.4	3	6.1	
Others	149	41.6	16	32.7	
NCI Risk					0.642
High	138	38.2	17	34	
Low	223	61.8	33	66	
Day 8 Prednisolone Response∧					0.804
Good	320	88.9	44	91.7	
Poor	40	11.1	4	8.3	
Day 33 PCR MRD∧					0.970
<0.01%	146	43.6	19	46.3	
0.01–1%	155	46.4	18	43.9	
≥1%	34	10	4	9.8	
PCR MRD Risk∧					0.966
Standard	134	38.4	17	37	
Intermediate	194	55.6	27	58.7	
High	21	6	2	4.3	
Ma-Spore Risk∧					0.463
Standard	109	30.2	14	28	
Intermediate	177	49	29	58	
High	75	20.8	7	14	
Ma-Spore Outcome					0.608
CCR	295	81.7	42	84	
Induction Failure	16	4.4	1	2	
Relapse	21	5.8	1	2	
Death	17	4.7	3	6	
Abandonment	12	3.3	3	6	
Race					<0.001
Chinese	156	43.2	35	70	
Malay	147	40.7	14	28	
Indian & Others	58	16.1	1	2	

Incidence of the *BIM* polymorphism is 50 out of 411 patients, or 12.2%. Abbreviations: Ma-Spore, Malaysia- Singapore; MRD, minimal residual disease; NCI, National Cancer Institute; PCR, polymerase chain reaction; CCR, continuous complete remission.

∧ indicates that data was unavailable for some patients.

**Figure 3:**
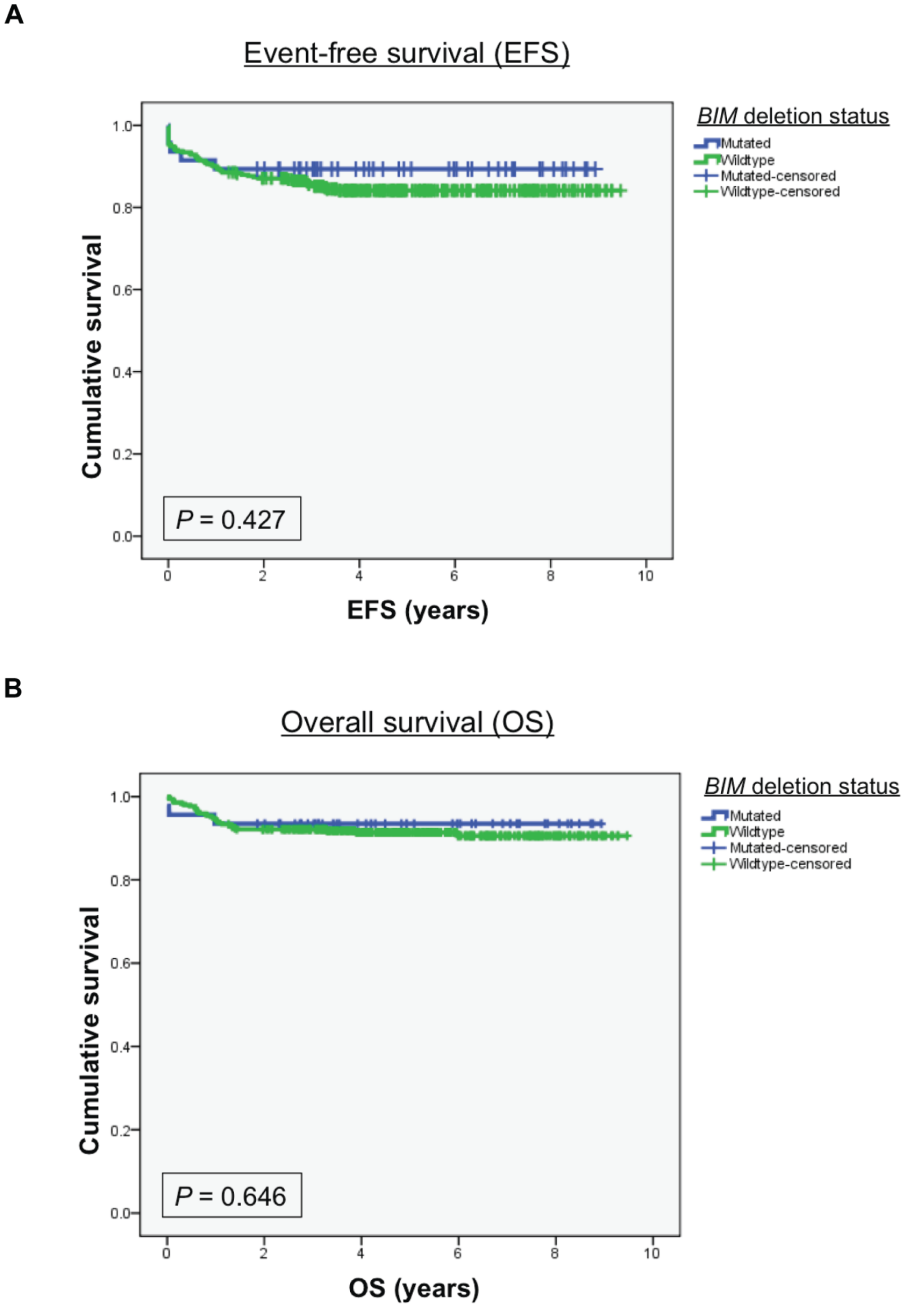
Retrospective analysis of the Ma-Spore ALL 2003 cohort according to the presence or absence of the *BIM* deletion polymorphism. Kaplan-Meier curves comparing event-free survival (**A**) or overall survival (**B**) in patients with or without the *BIM* deletion polymorphism are shown.

We next determined if the *BIM* deletion predicted for inferior outcomes at each of the three response assessment points described above. Here, and to our surprise, we found that there was no significant difference between patients with or without the deletion for GC response at day 8 (*P* = 0.804), MRD response at day 33 (*P* = 0.970), nor EFS (*P* = 0.427) or overall OS (*P* = 0.646) (Table 1, Figures 3A and 3B). Additionally, subgroup analysis by genetic subtype, race and risk category at diagnosis did not uncover any associations between the *BIM* deletion polymorphism and treatment outcome (data not shown). Together, these results demonstrate that the *BIM* deletion polymorphism does not predict for inferior outcomes following the administration of a modern GC-containing three-drug remission-induction regimen. Our clinical observations led us to propose that at least one or more of the chemotherapy agents employed during induction is able to overcome GC resistance conferred by the *BIM* deletion polymorphism.

### Chemotherapy overcomes GC resistance conferred by the *BIM* deletion polymorphism

To determine if any of the chemotherapy agents used in the induction regimen was able to overcome *BIM* deletion-mediated GC resistance, we treated the *BIM* deletion-containing clones with methotrexate, vincristine, and L-asparaginase individually, and in combination with dexamethasone. Cells were then assessed for activation of apoptotic cell death, BIM protein induction, and cell viability.

**Figure 4:**
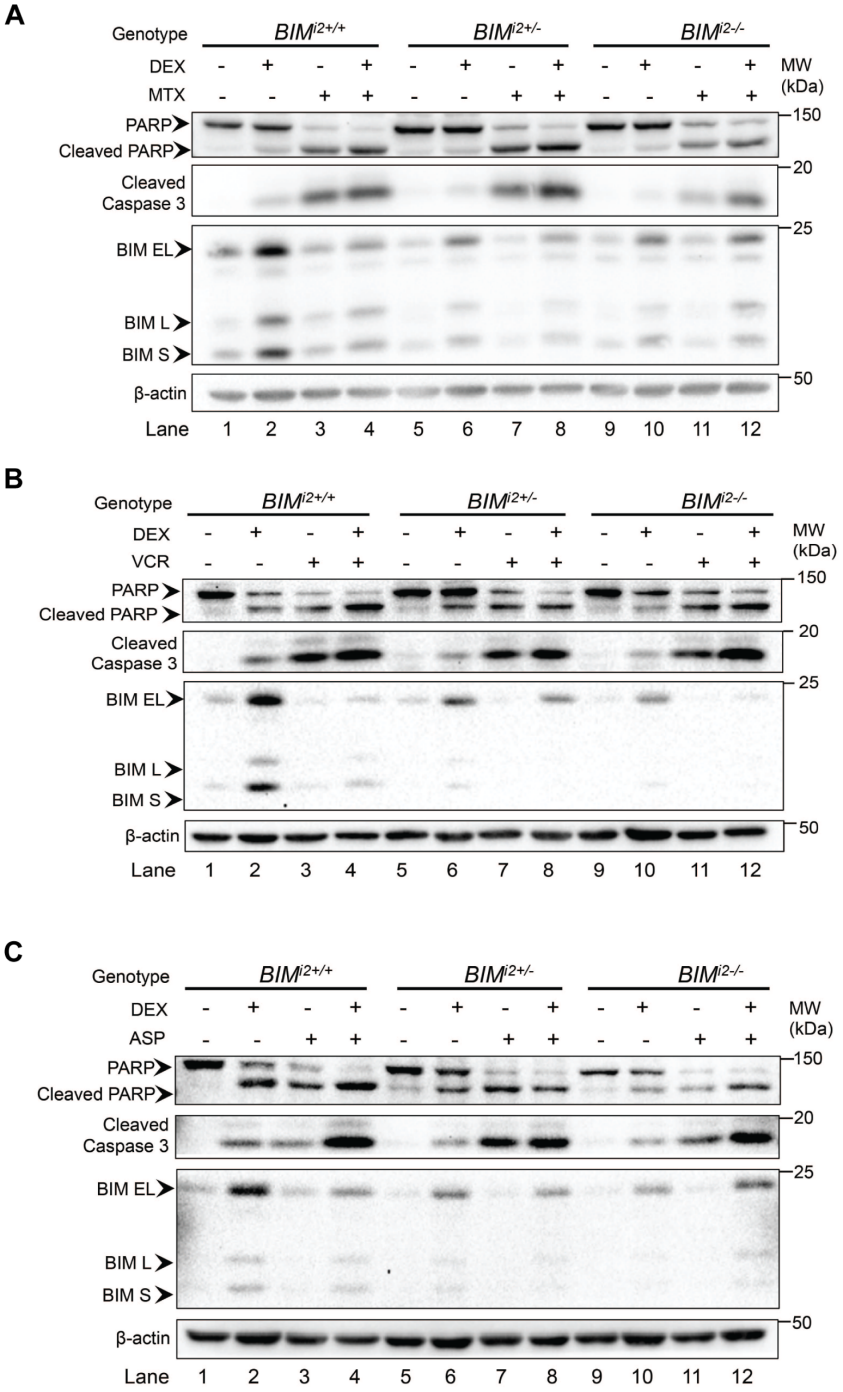
Methotrexate, vincristine, and L-asparaginase activate apoptosis in a BIM-independent manner, and overcome *BIM* deletion-mediated GC resistance. CCRF-CEM clones were treated with dexamethasone (DEX) (0.1 µM) with or without (**A**) methotrexate (MTX) (1 µM), (**B**) vincristine (VCR) (2 ng/ml), or (C) L-asparaginase (ASP) (0.5 IU/ml) for 48 h. Following incubation, cell lysates were obtained and analyzed for cleaved PARP and caspase 3, as well as BIM induction. β-actin was used as a loading control.

First, using immunoblot, we found that each of the three chemotherapy agents was individually able to induce equivalent levels of apoptotic cell death (as measured by the production of cleaved PARP and caspase 3) in the absence and presence of the *BIM* deletion (Figures 4A–C, compare lanes 3, 7 and 11). Importantly, we also observed that chemotherapy-induced apoptosis occurred without significant induction of any of the three BIM isoforms (BIM EL, L, and S) reported to be important for GC-induced apoptosis [31]. These results demonstrate that methotrexate, vincristine, and L-asparaginase are each able to induce ALL cell death in a BIM-independent manner, and that this occurred regardless of the *BIM* deletion status of the cell line.

**Figure 5:**
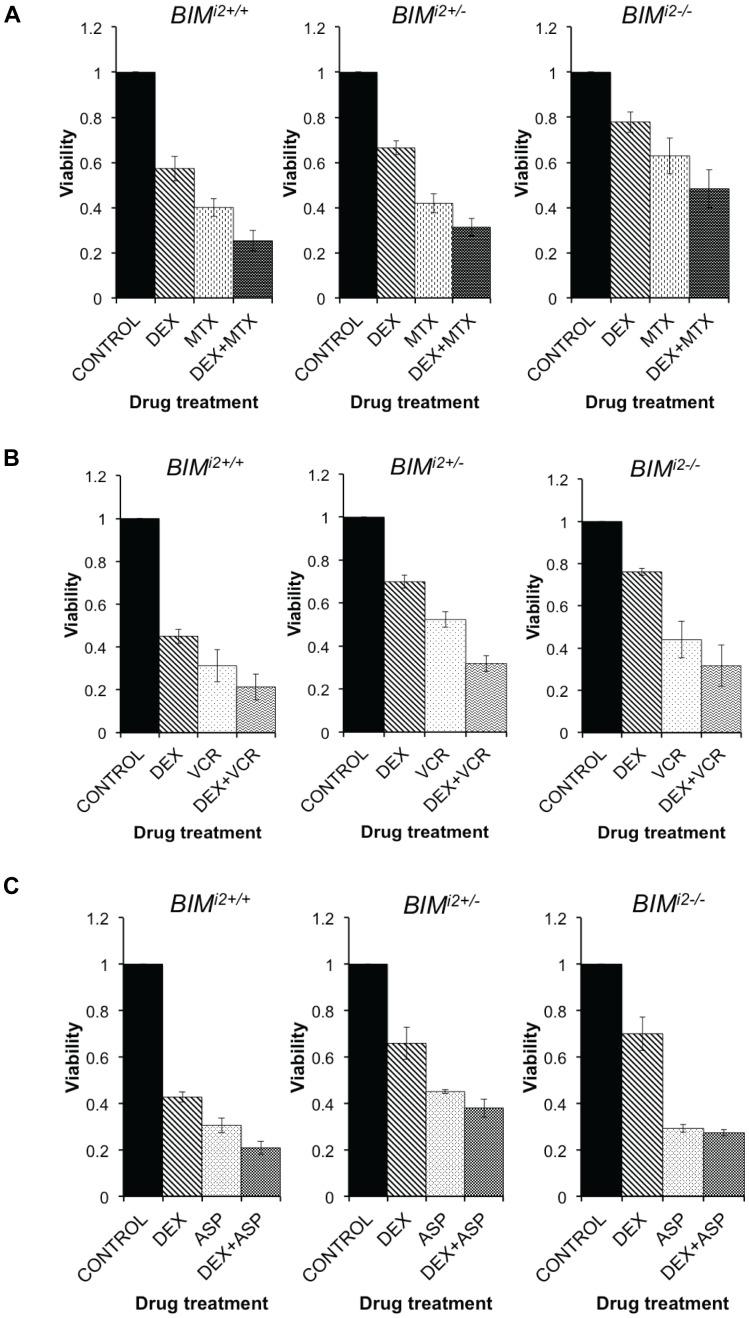
The addition of methotrexate, vincristine or L-asparaginase resensitizes *BIM* deletion-containing CCRF-CEM clones to dexamethasone. Cell viability was measured by MTS assay after (**A**) methotrexate (MTX) (1 µM), (**B**) vincristine (VCR) (2 ng/ml), or (**C**) L-asparaginase (ASP) (0.5 IU/ml) was used singly or in combination with dexamethasone (DEX) (0.1 µM) for 48 h. Values obtained for treated cells were normalized to the untreated control for the same genotype. Error bars indicate SEM (n = ) of 3 independent replicates.

Next, we found that when dexamethasone was combined with methotrexate, vincristine or L-asparaginase, there was a consistent increase in the level of activated PARP and caspase 3 compared to GC alone (Figures 4A–C, lanes 4, 8 and 12). Similarly, when cell viability was assayed, the addition of methotrexate (Figure 5A), vincristine (Figure 5B), or L-asparaginase (Figure 5C) to dexamethasone augmented cell death in deletion-containing clones. Taken together, our *in vitro* data suggest that the ability of the *BIM* deletion polymorphism to confer GC resistance can be overcome by the co-administration of several of the cytotoxic components of the Ma-Spore regimen, including methotrexate, vincristine, and L-asparaginase.

## Discussion

In the current work, we used a genome-editing approach to demonstrate that a common germline variant in the *BIM* gene is sufficient to confer GC resistance in ALL cell lines. Mechanistically, we confirm that cells harboring the deletion favor the splicing and expression of non-apoptotic isoforms of BIM, impairing the apoptotic response to GC exposure, and thereby promoting ALL cell survival. However, using a cohort of 411 uniformly-treated ALL patients, we also find that the deletion does not predict inferior responses to GC-containing multi-agent chemotherapy, and that this is associated with the ability of chemotherapy to induce BIM-independent cell death.

By generating CCRF-CEM subclones that were either wildtype, heterozygous or homozygous for the *BIM* polymorphism, we were able to demonstrate that the *BIM* deletion polymorphism is able to confer GC resistance in a T-ALL cell line. In these clones, the expected changes in splicing to favor the E3-containing, non-apoptotic splice variants were recapitulated in a polymorphism dose-dependent manner. We then showed that both upregulation of the E4-containing *BIM* isoforms and apoptosis upon GC treatment were impaired in the deletion-containing clones. Overall, our results are consistent with prior work demonstrating a critical role for BIM induction in GC-induced ALL cell death, particularly the EL, L, and S isoforms which harbor the E4- and BH3-containing isoforms capable of activating apoptosis [27], [31]. One limitation of our *in vitro* studies is the use of a single cell line, CCRF-CEM, which is a T-ALL line. Although we were unable to generate deletion-containing lines of other lineages, we expect that introduction of the deletion, which phenocopies a BIM knockdown of BIM, will likely confer glucocorticoid resistance in other ALL cell lines [27], [31], [32].

The inability of the *BIM* deletion to segregate poor versus good risk patients was somewhat surprising given previous reports describing the ability of *in vitro* as well as clinical GC responses to predict long-term outcomes in pediatric ALL [33]–[37]. Importantly, because we were able to generate isogenic cell lines with and without the *BIM* deletion, we could explore the mechanisms underlying our clinical observations. Here, we found that three other agents employed in the Ma-Spore ALL 2003 regimen, methotrexate, vincristine, and L-asparaginase, are each individually able to overcome *BIM* deletion-associated GC resistance. Our *in vitro* results also indicate that each of these drugs activate apoptotic cell death in a largely BIM-independent manner, and that this is likely to underlie their clinical efficacy in overcoming *BIM* deletion-mediated GC resistance. While the precise mechanisms by which methotrexate and L-asparaginase induce apoptosis remain ill-defined [43], it is interesting to note that the mechanism of vincristine-induced apoptosis has recently been described [44]. Here [44], and consistent with our observations, vincristine-induced apoptosis was shown to occur via the depletion of the pro-survival protein MCL1, a factor that has itself been shown to mediate GC resistance in ALL [45].

It is also important to highlight that *in vitro* resistance to GC, which has been shown to correlate with clinical responses [33], [34], may not necessarily readout for GC resistance *per se*. This is because such assays will also read out for more general mechanisms of resistance that would be expected to mediate cross-resistance among different drug classes, a conclusion that other studies have suggested [46], [47]. More recent work has also implicated other germline *BIM* variants in mediating drug resistance [48], [49]. Importantly, and reminiscent of our data, we note that it was the combination of a functional SNP in *BIM* with a SNP in the *MCL1* promoter (associated with increased *MCL1* expression) that best predicted OS in pediatric ALL [49], [50]. Together, our observations are consistent with a “BCL-2 rheostat” model where the cellular apoptotic threshold is set by the balance of pro-apoptotic BCL-2 family members such as BIM and anti-apoptotic members like MCL1 [27]. This model would predict that genetic variants affecting BCL2 family members may only be clinically important when two or more act in concert to alter the apoptotic threshold.

While there is increasing evidence that germline polymorphisms contribute to clinical heterogeneity in ALL [13]–[15], it is likely that only those variants capable of conferring alterations in biological behavior and/or multi-drug resistance will be associated with clinically meaningful endpoints. Thus, as we have demonstrated with the *BIM* deletion, polymorphisms that confer single-drug resistance in the setting of modern multi-agent ALL therapy are less likely to be of clinical importance. Indeed, variants that have been shown to predict poor response are enriched for genes expected to confer a multi-drug resistance phenotype, and include those that influence systemic drug clearance and intracellular drug concentrations [9], [12].

Finally, our observations also highlight the ability of at least three cytotoxic agents to induce apoptosis independently of BIM, and suggest that the success of modern day ALL regimens is due to the ability of individual agents to kill leukemia cells via targeting different components of the “BCL-2 rheostat”. Indeed, this general lesson may be applied to cancers where we have found that the *BIM* deletion does play a part in clinical drug resistance [17], and supports the use of judiciously chosen combination therapies to overcome *BIM* deletion-mediated drug resistance in these patients.

## References

[1] Mullighan CG (2012) The molecular genetic makeup of acute lymphoblastic leukemia. Hematology Am Soc Hematol Educ Program 2012: 389–396. 23233609 10.1182/asheducation-2012.1.389

[2] Pui CH, Evans WE (2006) Treatment of acute lymphoblastic leukemia. N Engl J Med 354: 166–178. 16407512 10.1056/NEJMra052603

[3] Locatelli F, Schrappe M, Bernardo ME, Rutella S (2012) How I treat relapsed childhood acute lymphoblastic leukemia. Blood 120: 2807–2816. 22896001 10.1182/blood-2012-02-265884

[4] Yeoh EJ, Ross ME, Shurtleff SA, Williams WK, Patel D, et al (2002) Classification, subtype discovery, and prediction of outcome in pediatric acute lymphoblastic leukemia by gene expression profiling. Cancer Cell 1: 133–143. 12086872 10.1016/s1535-6108(02)00032-6

[5] Martinelli G, Iacobucci I, Storlazzi CT, Vignetti M, Paoloni F, et al (2009) IKZF1 (Ikaros) deletions in BCR-ABL1-positive acute lymphoblastic leukemia are associated with short disease-free survival and high rate of cumulative incidence of relapse: a GIMEMA AL WP report. J Clin Oncol 27: 5202–5207. 19770381 10.1200/JCO.2008.21.6408

[6] Roberts KG, Morin RD, Zhang J, Hirst M, Zhao Y, et al (2012) Genetic alterations activating kinase and cytokine receptor signaling in high-risk acute lymphoblastic leukemia. Cancer Cell 22: 153–166. 22897847 10.1016/j.ccr.2012.06.005 PMC3422513

[7] McLeod HL (2013) Cancer pharmacogenomics: early promise, but concerted effort needed. Science 339: 1563–1566. 23539596 10.1126/science.1234139 PMC3900028

[8] Coate L, Cuffe S, Horgan A, Hung RJ, Christiani D, et al (2010) Germline genetic variation, cancer outcome, and pharmacogenetics. J Clin Oncol 28: 4029–4037. 20679599 10.1200/JCO.2009.27.2336

[9] Radtke S, Zolk O, Renner B, Paulides M, Zimmermann M, et al (2013) Germline genetic variations in methotrexate candidate genes are associated with pharmacokinetics, toxicity, and outcome in childhood acute lymphoblastic leukemia. Blood 121: 5145–5153. 23652803 10.1182/blood-2013-01-480335

[10] Xu H, Cheng C, Devidas M, Pei D, Fan Y, et al (2012) ARID5B genetic polymorphisms contribute to racial disparities in the incidence and treatment outcome of childhood acute lymphoblastic leukemia. J Clin Oncol 30: 751–757. 22291082 10.1200/JCO.2011.38.0345 PMC3295551

[11] Trevino LR, Shimasaki N, Yang W, Panetta JC, Cheng C, et al (2009) Germline genetic variation in an organic anion transporter polypeptide associated with methotrexate pharmacokinetics and clinical effects. J Clin Oncol 27: 5972–5978. 19901119 10.1200/JCO.2008.20.4156 PMC2793040

[12] Rocha JC, Cheng C, Liu W, Kishi S, Das S, et al (2005) Pharmacogenetics of outcome in children with acute lymphoblastic leukemia. Blood 105: 4752–4758. 15713801 10.1182/blood-2004-11-4544 PMC1895006

[13] Perez-Andreu V, Roberts KG, Harvey RC, Yang W, Cheng C, et al (2013) Inherited GATA3 variants are associated with Ph-like childhood acute lymphoblastic leukemia and risk of relapse. Nat Genet 45: 1494–1498. 24141364 10.1038/ng.2803 PMC4039076

[14] Yang JJ, Cheng C, Devidas M, Cao X, Campana D, et al (2012) Genome-wide association study identifies germline polymorphisms associated with relapse of childhood acute lymphoblastic leukemia. Blood 120: 4197–4204. 23007406 10.1182/blood-2012-07-440107 PMC3501717

[15] Yang JJ, Cheng C, Yang W, Pei D, Cao X, et al (2009) Genome-wide interrogation of germline genetic variation associated with treatment response in childhood acute lymphoblastic leukemia. JAMA 301: 393–403. 19176441 10.1001/jama.2009.7 PMC2664534

[16] Gaj T, Gersbach CA, Barbas CF 3rd (2013) ZFN, TALEN, and CRISPR/Cas-based methods for genome engineering. Trends Biotechnol 31: 397–405. 23664777 10.1016/j.tibtech.2013.04.004 PMC3694601

[17] Ng KP, Hillmer AM, Chuah CT, Juan WC, Ko TK, et al (2012) A common BIM deletion polymorphism mediates intrinsic resistance and inferior responses to tyrosine kinase inhibitors in cancer. Nat Med 18: 521–528. 22426421 10.1038/nm.2713

[18] Morel F, Ka C, Le Bris MJ, Herry A, Morice P, et al (2003) Deletion of the 5′ABL region in Philadelphia chromosome positive chronic myeloid leukemia: frequency, origin and prognosis. Leuk Lymphoma 44: 1333–1338. 12952226 10.1080/1042819031000097384

[19] Jabbour E, Cortes JE, Kantarjian HM (2009) Suboptimal response to or failure of imatinib treatment for chronic myeloid leukemia: what is the optimal strategy? Mayo Clin Proc 84: 161–169. 19181650 10.4065/84.2.161 PMC2664587

[20] Aichberger KJ, Mayerhofer M, Krauth MT, Vales A, Kondo R, et al (2005) Low-level expression of proapoptotic Bcl-2-interacting mediator in leukemic cells in patients with chronic myeloid leukemia: role of BCR/ABL, characterization of underlying signaling pathways, and reexpression by novel pharmacologic compounds. Cancer Res 65: 9436–9444. 16230407 10.1158/0008-5472.CAN-05-0972

[21] Bouillet P, Metcalf D, Huang DC, Tarlinton DM, Kay TW, et al (1999) Proapoptotic Bcl-2 relative Bim required for certain apoptotic responses, leukocyte homeostasis, and to preclude autoimmunity. Science 286: 1735–1738. 10576740 10.1126/science.286.5445.1735

[22] Cragg MS, Kuroda J, Puthalakath H, Huang DC, Strasser A (2007) Gefitinib-induced killing of NSCLC cell lines expressing mutant EGFR requires BIM and can be enhanced by BH3 mimetics. PLoS Med 4: 1681–1689 discussion 1690. 17973573 10.1371/journal.pmed.0040316 PMC2043013

[23] Gong Y, Somwar R, Politi K, Balak M, Chmielecki J, et al (2007) Induction of BIM is essential for apoptosis triggered by EGFR kinase inhibitors in mutant EGFR-dependent lung adenocarcinomas. PLoS Med 4: e294. 17927446 10.1371/journal.pmed.0040294 PMC2001209

[24] Kuribara R, Honda H, Matsui H, Shinjyo T, Inukai T, et al (2004) Roles of Bim in apoptosis of normal and Bcr-Abl-expressing hematopoietic progenitors. Mol Cell Biol 24: 6172–6183. 15226421 10.1128/MCB.24.14.6172-6183.2004 PMC434248

[25] Kuroda J, Puthalakath H, Cragg MS, Kelly PN, Bouillet P, et al (2006) Bim and Bad mediate imatinib-induced killing of Bcr/Abl+ leukemic cells, and resistance due to their loss is overcome by a BH3 mimetic. Proc Natl Acad Sci U S A 103: 14907–14912. 16997913 10.1073/pnas.0606176103 PMC1595449

[26] Erlacher M, Michalak EM, Kelly PN, Labi V, Niederegger H, et al (2005) BH3-only proteins Puma and Bim are rate-limiting for gamma-radiation- and glucocorticoid-induced apoptosis of lymphoid cells in vivo. Blood 106: 4131–4138. 16118324 10.1182/blood-2005-04-1595 PMC1895232

[27] Ploner C, Rainer J, Niederegger H, Eduardoff M, Villunger A, et al (2008) The BCL2 rheostat in glucocorticoid-induced apoptosis of acute lymphoblastic leukemia. Leukemia 22: 370–377. 18046449 10.1038/sj.leu.2405039 PMC4950962

[28] Wang Z, Malone MH, He H, McColl KS, Distelhorst CW (2003) Microarray analysis uncovers the induction of the proapoptotic BH3-only protein Bim in multiple models of glucocorticoid-induced apoptosis. J Biol Chem 278: 23861–23867. 12676946 10.1074/jbc.M301843200

[29] Schmidt S, Rainer J, Riml S, Ploner C, Jesacher S, et al (2006) Identification of glucocorticoid-response genes in children with acute lymphoblastic leukemia. Blood 107: 2061–2069. 16293608 10.1182/blood-2005-07-2853

[30] Bachmann PS, Piazza RG, Janes ME, Wong NC, Davies C, et al (2010) Epigenetic silencing of BIM in glucocorticoid poor-responsive pediatric acute lymphoblastic leukemia, and its reversal by histone deacetylase inhibition. Blood 116: 3013–3022. 20647567 10.1182/blood-2010-05-284968

[31] Abrams MT, Robertson NM, Yoon K, Wickstrom E (2004) Inhibition of glucocorticoid-induced apoptosis by targeting the major splice variants of BIM mRNA with small interfering RNA and short hairpin RNA. J Biol Chem 279: 55809–55817. 15509554 10.1074/jbc.M411767200

[32] Jiang N, Koh GS, Lim JY, Kham SK, Ariffin H, et al (2011) BIM is a prognostic biomarker for early prednisolone response in pediatric acute lymphoblastic leukemia. Exp Hematol 39: 321–329. 21130142 10.1016/j.exphem.2010.11.009

[33] Kaspers GJ, Pieters R, Van Zantwijk CH, Van Wering ER, Van Der Does-Van Den Berg A, et al (1998) Prednisolone resistance in childhood acute lymphoblastic leukemia: vitro-vivo correlations and cross-resistance to other drugs. Blood 92: 259–266. 9639525

[34] Den Boer ML, Harms DO, Pieters R, Kazemier KM, Gobel U, et al (2003) Patient stratification based on prednisolone-vincristine-asparaginase resistance profiles in children with acute lymphoblastic leukemia. J Clin Oncol 21: 3262–3268. 12947061 10.1200/JCO.2003.11.031

[35] Lauten M, Moricke A, Beier R, Zimmermann M, Stanulla M, et al (2012) Prediction of outcome by early bone marrow response in childhood acute lymphoblastic leukemia treated in the ALL-BFM 95 trial: differential effects in precursor B-cell and T-cell leukemia. Haematologica 97: 1048–1056. 22271901 10.3324/haematol.2011.047613 PMC3396677

[36] Dordelmann M, Reiter A, Borkhardt A, Ludwig WD, Gotz N, et al (1999) Prednisone response is the strongest predictor of treatment outcome in infant acute lymphoblastic leukemia. Blood 94: 1209–1217. 10438708

[37] Schrappe M, Arico M, Harbott J, Biondi A, Zimmermann M, et al (1998) Philadelphia chromosome-positive (Ph+) childhood acute lymphoblastic leukemia: good initial steroid response allows early prediction of a favorable treatment outcome. Blood 92: 2730–2741. 9763557

[38] Yeoh AE, Ariffin H, Chai EL, Kwok CS, Chan YH, et al (2012) Minimal residual disease-guided treatment deintensification for children with acute lymphoblastic leukemia: results from the Malaysia-Singapore acute lymphoblastic leukemia 2003 study. J Clin Oncol 30: 2384–2392. 22614971 10.1200/JCO.2011.40.5936

[39] Foley GE, Lazarus H, Farber S, Uzman BG, Boone BA, et al (1965) Continuous Culture of Human Lymphoblasts from Peripheral Blood of a Child with Acute Leukemia. Cancer 18: 522–529. 14278051 10.1002/1097-0142(196504)18:4<522::aid-cncr2820180418>3.0.co;2-j

[40] Stong RC, Korsmeyer SJ, Parkin JL, Arthur DC, Kersey JH (1985) Human acute leukemia cell line with the t(4;11) chromosomal rearrangement exhibits B lineage and monocytic characteristics. Blood 65: 21–31. 3917311

[41] Miyagi T, Ohyashiki J, Yamato K, Koeffler HP, Miyoshi I (1993) Phenotypic and molecular analysis of Ph1-chromosome-positive acute lymphoblastic leukemia cell lines. Int J Cancer 53: 457–462. 8428799 10.1002/ijc.2910530318

[42] Juan WC, Roca X, Ong ST (2014) Identification of cis-Acting Elements and Splicing Factors Involved in the Regulation of BIM Pre-mRNA Splicing. PLoS One 9: e95210. 24743263 10.1371/journal.pone.0095210 PMC3990581

[43] DeVita VT Jr, Lawrence TS, Rosenberg SA (2008) DeVita, Hellman, and Rosenberg's Cancer: Principles & Practice of Oncology. Philadelphia, PA, USA: Wolters Kluwer/Lippincott Williams & Wilkins. pp 427, 451, 490.

[44] Wertz IE, Kusam S, Lam C, Okamoto T, Sandoval W, et al (2011) Sensitivity to antitubulin chemotherapeutics is regulated by MCL1 and FBW7. Nature 471: 110–114. 21368834 10.1038/nature09779

[45] Wei G, Twomey D, Lamb J, Schlis K, Agarwal J, et al (2006) Gene expression-based chemical genomics identifies rapamycin as a modulator of MCL1 and glucocorticoid resistance. Cancer Cell 10: 331–342. 17010674 10.1016/j.ccr.2006.09.006

[46] Pieters R, Kaspers GJ, van Wering ER, Huismans DR, Loonen AH, et al (1993) Cellular drug resistance profiles that might explain the prognostic value of immunophenotype and age in childhood acute lymphoblastic leukemia. Leukemia 7: 392–397. 8445945

[47] Kaspers GJ, Pieters R, Van Zantwijk CH, Van Wering ER, Veerman AJ (1995) Clinical and cell biological features related to cellular drug resistance of childhood acute lymphoblastic leukemia cells. Leuk Lymphoma 19: 407–416. 8590840 10.3109/10428199509112198

[48] Augis V, Airiau K, Josselin M, Turcq B, Mahon FX, et al (2013) A Single Nucleotide Polymorphism in cBIM Is Associated with a Slower Achievement of Major Molecular Response in Chronic Myeloid Leukaemia Treated with Imatinib. PLoS One 8: e78582. 24223824 10.1371/journal.pone.0078582 PMC3818406

[49] Gagne V, Rousseau J, Labuda M, Sharif-Askari B, Brukner I, et al (2013) Bim polymorphisms: influence on function and response to treatment in children with acute lymphoblastic leukemia. Clin Cancer Res 19(18): 5240–5249. 23908358 10.1158/1078-0432.CCR-13-1215 PMC4128417

[50] Sanchez R, St-Cyr J, Lalonde ME, Healy J, Richer C, et al (2013) Impact of promoter polymorphisms in key regulators of the intrinsic apoptosis pathway in childhood acute lymphoblastic leukemia outcome. Haematologica. doi: 10.3324/haematol.2013.085340 10.3324/haematol.2013.085340 PMC3912962 24038028

